# Adhesion and Contact Aging of Acrylic Pressure-Sensitive Adhesives to Swollen Elastomers

**DOI:** 10.1021/acs.langmuir.3c03413

**Published:** 2024-02-15

**Authors:** Anushka Jha, Stefan Gryska, Carlos Barrios, Joelle Frechette

**Affiliations:** † Chemical and Biomolecular Engineering, Johns Hopkins University, Baltimore, Maryland 21218, United States; ‡ 3M Center, 3M Company, Building 201-4N-01, St. Paul, Minnesota 55144-1000, United States; § Carlos Barrios Consulting LLC, Frisco, Texas 75034, United States; ∥ Chemical and Biomolecular Engineering, University of California, Berkeley, California 94720, United States; ⊥ Energy Technology Area, Lawrence Berkeley National Laboratory, Berkeley, California 94720, United States

## Abstract

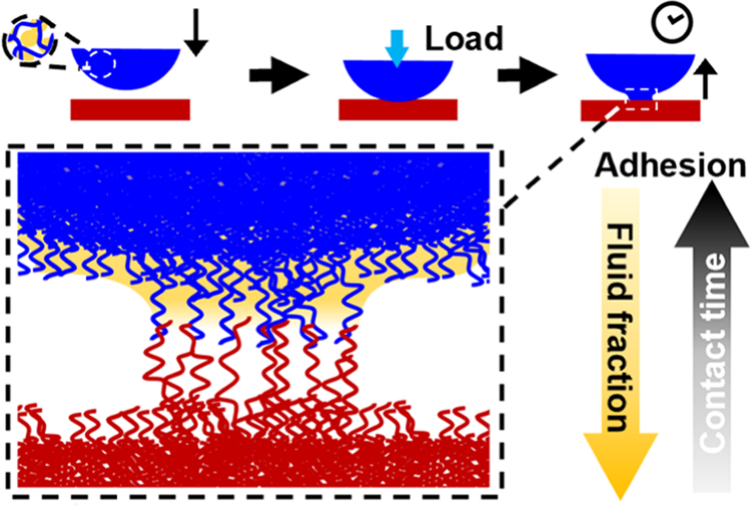

Fluid-infused (or swollen) elastomers are known for their antiadhesive properties. The presence of excess fluid at their surface is the main contributor to limiting contact formation and minimizing adhesion. Despite their potential, the mechanisms for adhesion and contact aging to fluid-infused elastomers are poorly understood beyond contact with a few materials (ice, biofilms, glass). This study reports on adhesion to a model fluid-infused elastomer, poly(dimethylsiloxane) (PDMS), swollen with silicone oil. The effects of oil saturation, contact time, and the opposing surface are investigated. Specifically, adhesion to two different adherents with comparable surface energies but drastically different mechanical properties is investigated: a glass surface and a soft viscoelastic acrylic pressure-sensitive adhesive film (PSA, modulus ∼25 kPa). Adhesion between the PSA and swollen PDMS [with 23% (w/w) silicone oil] retains up to 60% of its value compared to contact with unswollen (dry) PDMS. In contrast, adhesion to glass nearly vanishes in contact with the same swollen elastomer. Adhesion to the PSA also displays stronger contact aging than adhesion to glass. Contact aging with the PSA is comparable for dry and unsaturated PDMS. Moreover, load relaxation when the PSA is in contact with the PDMS does not correlate with contact aging for contact with the dry or unsaturated elastomer, suggesting that contact aging is likely caused by chain interpenetration and polymer reorganization within the contact region. Closer to full saturation of the PDMS with oil, adhesion to the PSA decreases significantly and shows a delay in the onset of contact aging that is weakly correlated to the poroelastic relaxation of the elastomer. Additional confocal imaging suggests that the presence of a layer of fluid trapped at the interface between the two solids could explain the delayed (and limited) contact aging to the oil-saturated PDMS.

## Introduction

Swollen elastomers are a class of liquid-infused soft materials gaining popularity for their antiadhesive properties in various applications ranging from durable icephobic coatings^1−4^ to antibiofouling surfaces.^5,6^ A key feature of liquid-infused elastomers is the presence of a liquid at the surface. This fluid film greatly reduces adhesion to highly adhesive substances such as mussel foot proteins,^5,7−9^ and limits the growth of bacterial films.^10,11^ A second key feature of fluid-infused elastomers is that the elastomer network acts as a reservoir to maintain long-term surface coverage of fluids to prevent adhesion under harsh conditions.^2,11,12^ Both ice adhesion and biofilm formation on fluid-infused surfaces are commonly investigated by growing a layer of ice (or biofilm) on the swollen elastomers prior to detachment.^3,10,13,14^ However, there are limited studies investigating how adhesive contact is made to swollen elastomers and, more importantly, how adhesion evolves with contact time or fluid content.

The presence of fluid at the surface of a swollen elastomer reduces adhesion with rigid substrates at short contact times.^15^ The amount of fluid present at the surface depends on the fluid content in the bulk, and so do the adhesive properties.^5,13,15^ In addition, for ultrasoft swollen elastomeric gels, where Young’s modulus is *E* ∼ *O*(1) kPa, fluid separates from the bulk and forms a wetting ridge at the contact line with another solid surface.^16^ For example, Jensen et al. studied contact between a silica microsphere and a swollen ultrasoft elastomer and showed fluid separation, i.e., the fluid being displaced from the bulk solid to the triple contact line, caused by the stress gradient developed in the contact region.^16^ In addition, Cai et al.^17^ also showed that the amount of fluid separated near the triple contact line of a swollen elastomer–water droplet interface is a nonmonotonic function of the bulk fluid fraction. They determined that the extent of fluid separation arises from a balance between elastic stresses in the gel and the osmotic pressure difference. For stiffer swollen gels (or swollen elastomers) in contact with a rigid surface, fluid at the interface prevents the formation of solid–solid contact and leads to a decrease in adhesion.^15^ With more compliant substances such as mussel plaques, the adhesion protein secreted by the mussel foot may be unable to displace excess lubricant present at the surface of an infused elastomer, thus limiting adhesion.^8^ However, live mussel species are highly complex as they have intrinsic mechanosensors for solid surfaces that coexist with the other physical processes, such as wetting, that would contribute to adhesion.^5^ Despite all of these investigations, there is a lack of understanding of the mechanisms for adhesion and contact formation to swollen elastomers with other polymeric-compliant materials, especially those with high surface energy, such as adhesives. Specifically, as a limiting case, whether or not soft adhesives such as pressure-sensitive adhesives (PSAs) can displace fluid or seek a solid surface (akin to mussel feet) present at the elastomer/air interface to form an adhesive bond remains to be investigated.

Contact time plays an important role in increasing adhesion between two solid surfaces, also described as “contact aging”. A number of mechanisms can contribute to the contact aging of dry elastomers, such as slow contact creep and relaxation, polymer reorganization near the interface, or chain interpenetration and diffusion.^18−24^ The theory of muco-adhesion,^24,25^ which looks at adhesion with mucosal surfaces, includes chain interpenetration as one of the key mechanisms enhancing adhesion over time. It has been suggested that the transport of water across the mucin–hydrogel interface can facilitate chain interpenetration and increase adhesion over time.^26^ In addition, fluid-filled networks such as hydrogels undergo poroelastic relaxation under a deformation field, which may also lead to fluid transport away from the interface, enhancing dry contact and thus increasing adhesion.^27^ We have shown previously that antiadhesive performance between poly(dimethylsiloxane) (PDMS) swollen with silicone oil and glass is maintained over the poroelastic time scale.^15^ It is possible for the fluid layer at the interface to prevent adhesion to be overcome with time and compromise the antiadhesive property when a swollen elastomer is in contact with another permeable and compliant polymer.

**Figure 1:**
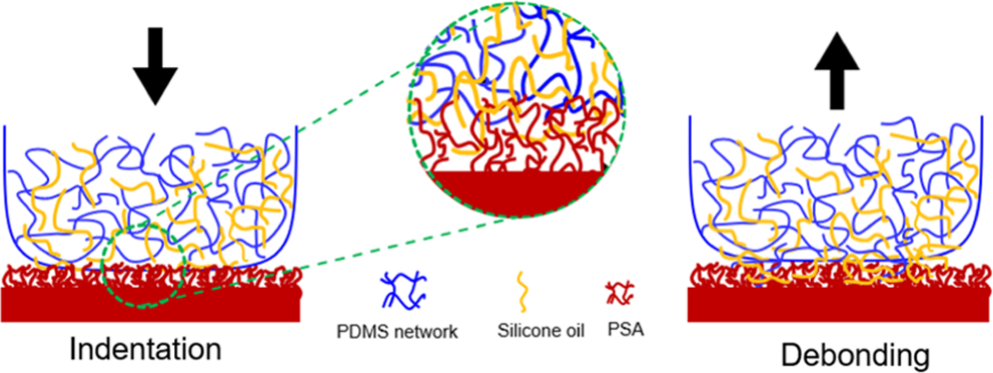
Schematic for contact between swollen PDMS and PSA.

In this work, we investigate adhesion between a model swollen elastomer and an acrylic pressure-sensitive adhesive (Figure 1). Throughout, adhesion measurements are compared to the adhesion of the same swollen elastomers with glass. We find that, in comparison to glass, the PSA is able to maintain good solid–solid contact at very high oil content at short contact times. Two different contact loads are applied to the PDMS-probe system. We observe that higher contact load affects the magnitude of adhesion of swollen PDMS with the PSA but not with glass, indicative of increased solid–solid contact with the compliant PSA. We then investigate how the oil fraction within the swollen elastomer affects the onset and extent of contact aging between the elastomer and PSA. We found that the amount of oil at the surface of the swollen PDMS plays a key role in delaying contact aging.

## Materials and Methods

### Materials

Sylgard 184 is a two-part silicone elastomer kit that was used for fabricating the elastomer samples. The elastomer was swollen in silicone oil (Gelest, Inc., 10 cSt, trimethylsiloxy terminated). ACS-grade chemicals were sourced from Sigma-Aldrich. Samples were used within 1 day after preparation. Poly(2-ethyl-hexyl acrylate)-*co*-acrylic-acid pressure-sensitive adhesive was provided by 3M Company.^28,29^

### PDMS Lens Fabrication and Swelling

The PDMS base was prepared by curing a 4 mm thick slab of 10:1 Sylgard 184 at 75 °C at 1 atm pressure for 16 h in a vacuum oven. Flat disks with a diameter of 6 mm were punched out using a biopsy punch. For hemispherical PDMS lenses, 40 μL of the 10:1 Sylgard mixture was drop cast on the precured base and cured again for 16 h at 75 °C and 1 atm pressure. PDMS samples were soaked in *n*-hexane for 6 h to extract the unreacted oligomers. Excess *n*-hexane was replaced by soaking the samples in ethanol for ∼30 min in a sonicator bath. After the removal of ethanol from the extracted lenses by heating the samples at 75 °C at 1 atm pressure for 16 h, the final dry weight was recorded as *m*_i_. Samples were swollen in silicone oil according to the protocol mentioned in our previous work,^15^ and the final weight was recorded as *m*_f_. The fraction of oil in the elastomer matrix was then calculated as ϕ = (*m*_f_ – *m*_i_)/*m*_i_. For 10:1 Sylgard, the maximum fraction of oil in the elastomer is ϕ_max_ = 0.4, which is in agreement with literature values for the same elastomer–oil combination.^11^ Henceforth, we will use ϕ̂ = ϕ/ϕ_max_ as a measure of the extent of swelling in the swollen elastomer. The elastomers were swollen over the entire range of swelling 0 ≤ ϕ̂ < 1.

### PSA

A model acrylic PSA consisting of 2-ethylhexyl acrylate and acrylic acid as comonomers was synthesized at 3M Company and supplied as 25 μm thick sheets on a PET release liner. A detailed account of the synthetic process, composition, and physical characterization of the PSA is given in the study by Karnal et al.^28,29^ PSA samples were prepared for adhesion measurements and imaging by cutting out samples of size 10 mm × 15 mm. The sample was gently applied to a clean glass slide. To ensure even contact with the glass slide, a 2 kg roller was run over the PET lining of the sample ∼20 times, and the lining was then removed.

### Constant Load and Indentation Measurements

A custom-built multifunctional force microscope (MFM) was used to conduct indentation, relaxation, and adhesion measurements in a sphere-plane geometry.^30^ The hemispherical swollen lenses were glued on a glass window using an ultrathin layer of uncured 10:1 Sylgard 184 and mounted on a cantilever spring with spring constant *k*_spring_ = 4500 N/m. The dry/swollen lens was lowered into contact with the PSA via a microcontroller, and the deflection Δ*x* in the cantilever spring was measured by a fiber optic (FO) sensor. A home-built LabView data acquisition program was used to record the motor movement Δ*M*, FO sensor reading, and convert the deflection into a force *F* = *k*_spring_Δ*x*. Images of the contact region were also acquired, and their timestamp information was logged in the program. The MFM allows us to control the velocity of approach and retraction and set a constant contact force via a force-feedback loop that controls the motor velocity during dwell to maintain the dwell force at the desired set point.

Contact and adhesion between the PDMS lens and PSA film were investigated for two different loading conditions: (1) constant load and (2) constant indentation depth. For constant load measurements, the spring was lowered to bring the PDMS lens into contact at a speed of *v* = 50 μm/s, and the contact load was maintained at a set point of *F* = 10 mN for different contact times (100 s ≤ *t* ≤ 22 × 10^3^ s) using a force-feedback control loop. The motor velocity was maintained at 50 μm/s to adjust the motor position and maintain the load set point. After a set dwell time, the spring was retracted to detach the spherical probe from the planar surface (PSA or glass) at a constant speed of *v* = 50 μm/s. For constant indentation measurements, the PDMS lens was brought into contact with the PSA at 500 μm/s to apply a step-indentation. The lens was then retracted at 50 μm/s after set contact time. Each contact and adhesion measurement was repeated thrice to ensure the reproducibility of data.

### Confocal Microscopy

**Figure 2:**
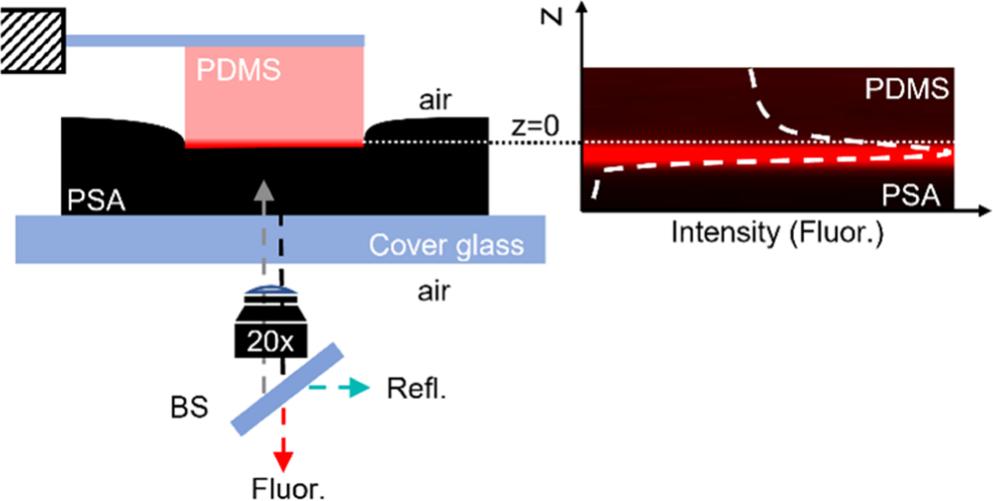
Imaging oil fluorescence in contact between a PDMS cube and a PSA film. Schematic, not to scale. The bright red layer below the PDMS layer is due to the fluorescence signal of dyed silicone oil detected within the PSA.

A confocal laser scanning microscope (Olympus FV3000) with a 20x objective was used to image the silicone oil at the PDMS–PSA interface. Nile red was chosen as a fluorescent dye to tag silicone oil due to its lipophilicity and a wide gap between the excitation (ex) and emission (em) spectra.^31^ A 0.5 mM solution of Nile red in silicone oil was obtained by mixing 3.18 mg dye (10^–5^ moles) in 1 mL of acetone and dispersing the solution in 20 mL of silicone oil. The stained oil solution was further diluted to get the desired concentration of 0.1 mM Nile red in silicone oil and stored in a glass vial. The vial was capped and agitated to disperse the dye uniformly in silicone oil. The stained dye solution was then used to swell dry (extracted) flat PDMS cubes of size 4 mm × 4 mm × 4 mm to the desired saturation. The PSA sample was placed on the sample stage, and light reflected from the PSA–air interface was used to determine the free surface of the PSA (see Figure S1) and position the objective accordingly. A PDMS cube mounted on a thin glass cantilever was lowered onto the PSA (Figure 2) and glass (Figure S2) surface at a speed of 50 μm/s in the vertical direction using a motorized stage. A 514 nm diode laser was used to excite the fluorescent dye, and the emitted signal was collected at 561 nm. See the Supporting Information for the ex/em spectra for the swollen PDMS. The imaging software scans ∼5 μm above the PSA surface and up to a depth of 35 μm in the *z*-direction with a *z*-resolution of ∼0.8 μm. We started acquiring data before the two surfaces came in contact (*t* = 0) and continued scanning until *t* = 1000 s with a resolution of ∼5 s (time to acquire a full *z*-stack). Data were then exported to and analyzed using ImageJ.

## Results and Discussion

### Adhesion after “Short” Contact Times: Dependence on the Oil Fraction

**Figure 3:**
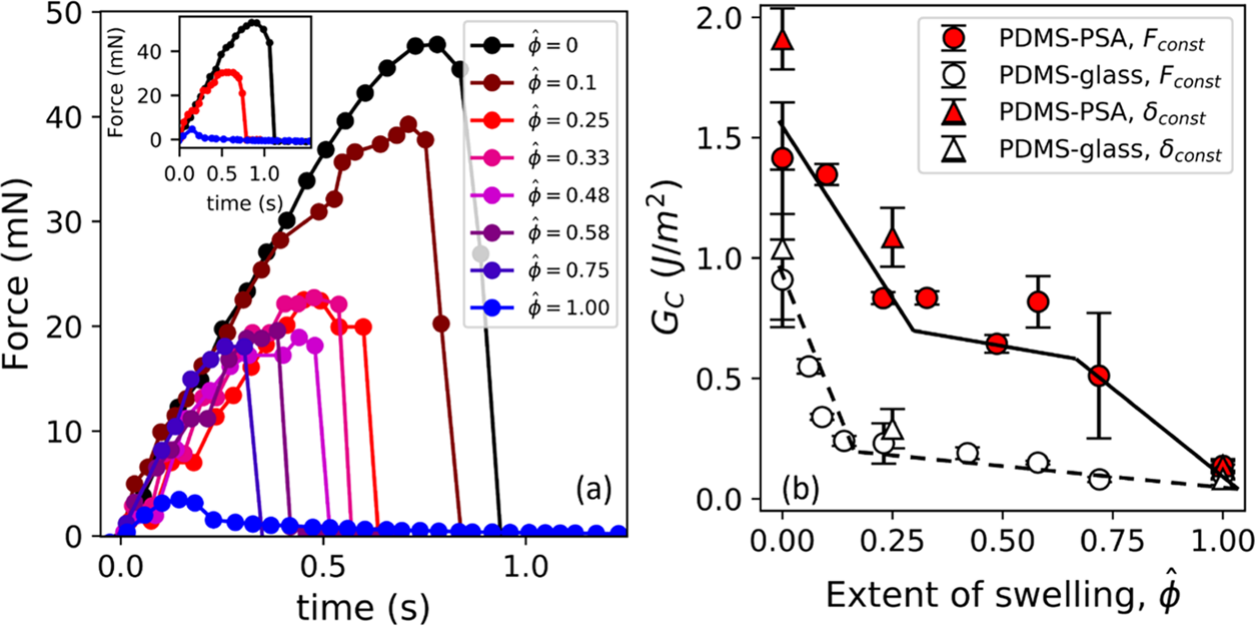
Decrease in adhesion with an increase in the oil content. (a) Representative force vs time curves during detachment of a PDMS lens from the PSA film after being in contact for 100 s at a constant load of 10 mN across the full range of oil fractions (ϕ̂ = ϕ/ϕ_max_). The inset shows force vs time for detachment after a constant indentation depth of ∼37 μm. (b) Critical strain energy release rate as a function obtained from the data in (a) (filled, red) and also for adhesion between the same PDMS probes and a glass surface (empty symbols). Circles and triangles denote constant load and constant indentation depth, respectively. Dashed and solid lines in (b) are to guide the eye. Force vs time curves in (a) are representative curves selected from a set of 3 repeats reported in Figure S3. Error bars in (b) represent the standard error of the mean.

Even a small amount of oil in the PDMS affects its adhesion to PSA (Figure 3). We made contact between a spherical PDMS probe and a thin (25 μm thickness) PSA film and kept the surfaces in contact at a constant load (*F* = 10 mN) for *t*_C_ = 100 s prior to detachment. We chose a contact time of 100 s based on the time scale for viscoelastic relaxation of the contact between PDMS (without oil) and glass.^15^ The bulk relaxation of the PSA film does not have a significant contribution to the overall relaxation of the PDMS–PSA contact (Figures S4 and S5). We refer to contact times of *t*_C_ = 100 s as a “short contact time”. For comparison, we also measured the detachment force after 100 s at a constant indentation depth of δ = 37 μm for ϕ̂ = 0, 0.25, and 1 (Figure 3a, inset). From the adhesion measurements, we estimate the critical strain energy release rate, *G*_c_, using the JKR relationship^32^*F*_max_ = 3/2π*RG*_c_, where *F*_max_ is the maximum tensile force during retraction (also referred to henceforth as the adhesive strength) and *R* ∼ 6 mm is the radius of curvature of the PDMS lens.

**Figure 4:**
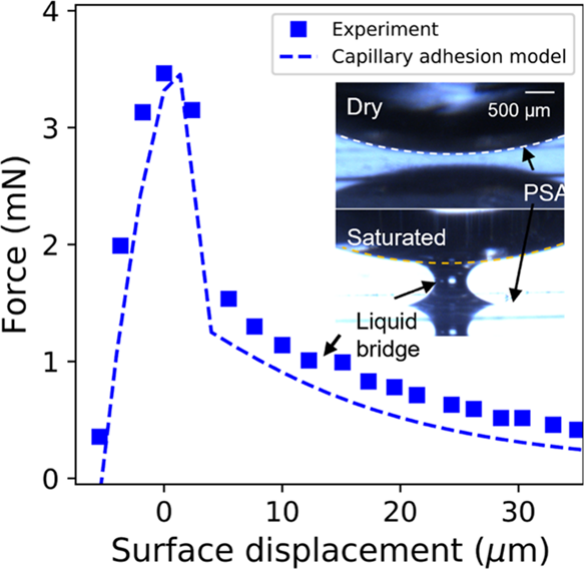
Representative force vs time during debonding of fully swollen PDMS from PSA after contact at constant load. The inset shows side-view images during the debonding of dry (ϕ̂ = 0) and saturated (ϕ̂ = 1) PDMS from PSA. The liquid capillary bridge is seen in both the force curve and in the side-view images during the debonding of saturated PDMS and is absent for dry PDMS.

We observe that the adhesive strength *F*_max_ decreases with increasing oil fraction in the PDMS matrix (ϕ̂), indicating poorer adhesion as the oil content increases (Figure 3a). We also observe a similar trend in *F*_max_ with increasing ϕ̂ after contact at constant indentation depth. Additionally, we observe that the debonding curves have similar characteristics for a range of oil content below saturation (i.e., ϕ̂ < 1), also indicating a similar debonding mechanism. At ϕ̂ = 1 (at saturation), we see a tail in the force versus time curve beyond *F*_max_ (Figure 4). We have seen capillary bridge formation in 1 of 3 repeat measurements for ϕ = 0.75. This indicates that the amount of oil at the interface likely transitions from not sufficient to enough to form a bridge observable in our experiments between ϕ = 0.75 and 1. The elastomer is saturated with oil, and this tail in the force vs time curve is caused by the presence of a liquid capillary bridge formed between the surfaces during debonding.^33^ This capillary bridge is visible from side-view imaging and contributes to overall adhesion with the fully swollen PDMS (Figure S6). We also compare the critical strain energy release rate for PSA–PDMS adhesion to glass–PDMS adhesion for the same applied (and constant) load and constant indentation depth during contact. Note that while the trend of decreasing *G*_C_ with ϕ̂ is independent of the loading conditions, the magnitude of *F*_max_ (and therefore *G*_C_) is higher for constant indentation depth with an initial load *F* ∼ 70 mN. Our result showing a decrease in adhesion is consistent with our previous study on the adhesion of the same swollen elastomers with glass and other prior studies on ice adhesion reported in the literature.^3,13,15^ We observe that for the same swelling ratio and loading conditions, the PSA–PDMS contact maintains stronger adhesion (relative to their dry counterpart) than glass–PDMS (Figure 3b). In Figure 3b, it can also be seen that for PDMS–PSA adhesion, *G*_C_ drops by 40% of its dry value, i.e., from 1.41 J/m^2^ at ϕ̂ = 0 to 0.83 J/m^2^ at ϕ̂ = 0.25. As a comparison, for the same oil saturation (ϕ̂ = 0.25), *G*_C_ for glass–PDMS adhesion drops by ∼70% of its dry value, i.e., from 0.91 to 0.23 J/m^2^. However, at complete saturation, the values of *G*_*C*_ are similar for PDMS–PSA and PDMS–glass adhesion, 0.14 and 0.10 J/m^2^, respectively, which is very close to the value of *G*_cap_ = 0.13 J/m^2^ calculated using the capillary adhesion model.^15^ Stronger debonding is attributed to the formation of better conformal contact during loading with a soft viscoelastic PSA.^34^ In contrast to a rigid substrate like glass, PSA is able to maintain stronger adhesion with swollen PDMS over a wider range of oil fractions.

**Figure 5:**
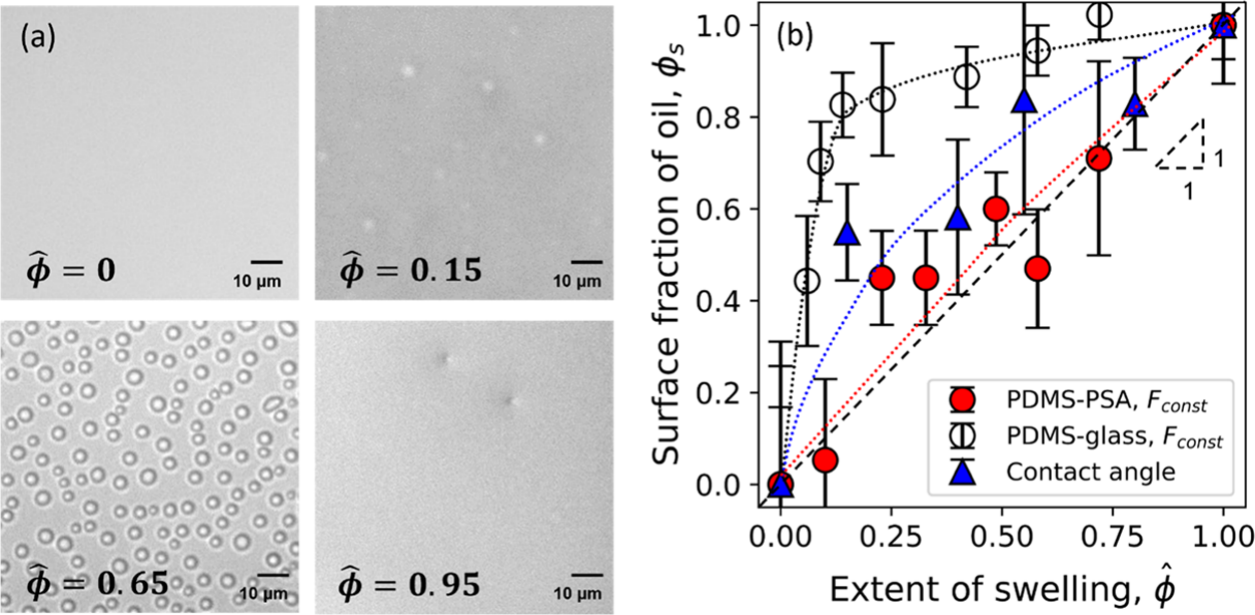
Visualizing the surface of PDMS before and after contact. (a) Images of the free surface of swollen PDMS at different ϕ̂. The density of oil droplets (circular patches) increases with increasing ϕ̂. At ϕ̂ = 0.95, interference rings around defects indicate the presence of fluid. (b) The effective surface fraction of oil at the swollen PDMS surface in contact with PSA (red circle) and glass (empty circle) and from contact angle measurements (blue triangle). Dashed lines are to guide the eye.

Images of the swollen PDMS surface obtained using reflected bright-field microscopy show the presence of droplets at the air–swollen elastomer interface (Figure 5a). The formation of these droplets is due to a wetting instability known as autophobic dewetting^35−37^ and has been discussed in our prior work.^15^ As the oil content in the elastomer increases, the amount of oil at the interface also increases. The surface energy of the swollen elastomer γ_tot_ (measured in our prior work^15^ using the two-liquid OWRK method)^38^ increases with increasing ϕ̂. Assuming a Cassie–Baxter type relationship^39,40^ between the area fraction of oil and dry elastomer and the surface energy, we can obtain the effective surface coverage of oil ϕ_s_ = (γ_tot_ – γ_dry_)/(γ_oil_ – γ_dry_), where γ_dry_ ∼ 17 mJ/m^2^ is the surface energy of dry elastomer and γ_oil_ ∼ 20.1 mJ/m^2^ is the surface tension of oil (Gelest, Inc.). We extend the Cassie–Baxter relationship for heterogeneous surfaces to apply to the swollen surface in contact with PSA. We assume that the mixing rule based on the area fraction would also apply to adhesion, i.e., the strain energy release rate between the two surfaces, eq 1. While this is a highly simplified approximation (especially with fluid present on the surface), we make this assumption since the strain energy release rate *G*_C,oil_ = 0.14 ± 0.02 J/m^2^ for fully swollen PDMS–PSA adhesion is negligible compared to dry PDMS–PSA adhesion, *G*_C,dry_ = 1.41 ± 0.23 J/m^2^.
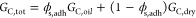
1where *G*_C,tot_, *G*_C,dry_, and *G*_C,oil_ are the strain energy release rate during debonding from the swollen, dry (ϕ̂ = 0), and fully swollen (ϕ̂ = 1) elastomer, respectively. ϕ_s_ and ϕ_s,adh_ for glass–PDMS and PSA–PDMS adhesion have been plotted in Figure 5b.

We use the effective area fraction ϕ_S_ as a tool to estimate the surface oil fraction on the PDMS in air and in contact with the PSA or glass. Based on this analysis, we found that the surface fraction of the PDMS covered by the oil (blue triangles in Figure 5b) is comparable to the estimated interfacial oil trapped at the PSA–PDMS interface (red circles in Figure 5b). In contrast, the PDMS–Glass interface appears to have excess oil trapped in the contact region. This similarity in the surface oil fraction (PSA–PDMS contact and contact angle on swollen PDMS) suggests that the PSA can access nearly all of the initially available solid surface on the swollen PDMS and form intimate solid–solid contact (at short contact time). This is in line with the ability of pressure-sensitive adhesives to deform and form conformal contact with other surfaces.^34,41^ The glass–PDMS interface, on the other hand, appears to have more oil coverage, even at low ϕ̂. We suspect that the higher apparent oil fraction for the PDMS–glass contact is due to the flattening (spreading) of trapped oil droplets in the contact region.^15^ It is also possible for hydrodynamic drainage to remove excess fluid from the interface and allow solid–solid contact to form between the PDMS and another surface. The initial fluid film thickness on the swollen PDMS surface can range from 0.4 to 4 μm for similar elastomer–oil combinations based on measurements reported in the literature.^5,9,10^ Based on the Stefan–Reynolds model,^42,43^ we found that the time required for a 0.4–4 μm thick layer to reduce to 50 nm would be ∼ O(1000) s. The time scale for fluid drainage is much slower than the contact time *t* = 100 s, and, therefore, we do not expect fluid drainage to play an important role in affecting swollen PDMS adhesion at short contact times. For contact between the swollen PDMS and the PSA, the fluid content at the interface could also be altered via diffusion of oil into the PSA. However, the low solubility of silicone oil in acrylic PSAs, owing to the difference in their respective solubility parameters,^44,45^ suggests that diffusion of oil in the PSA would be largely unfavorable/very slow.

**Figure 6:**
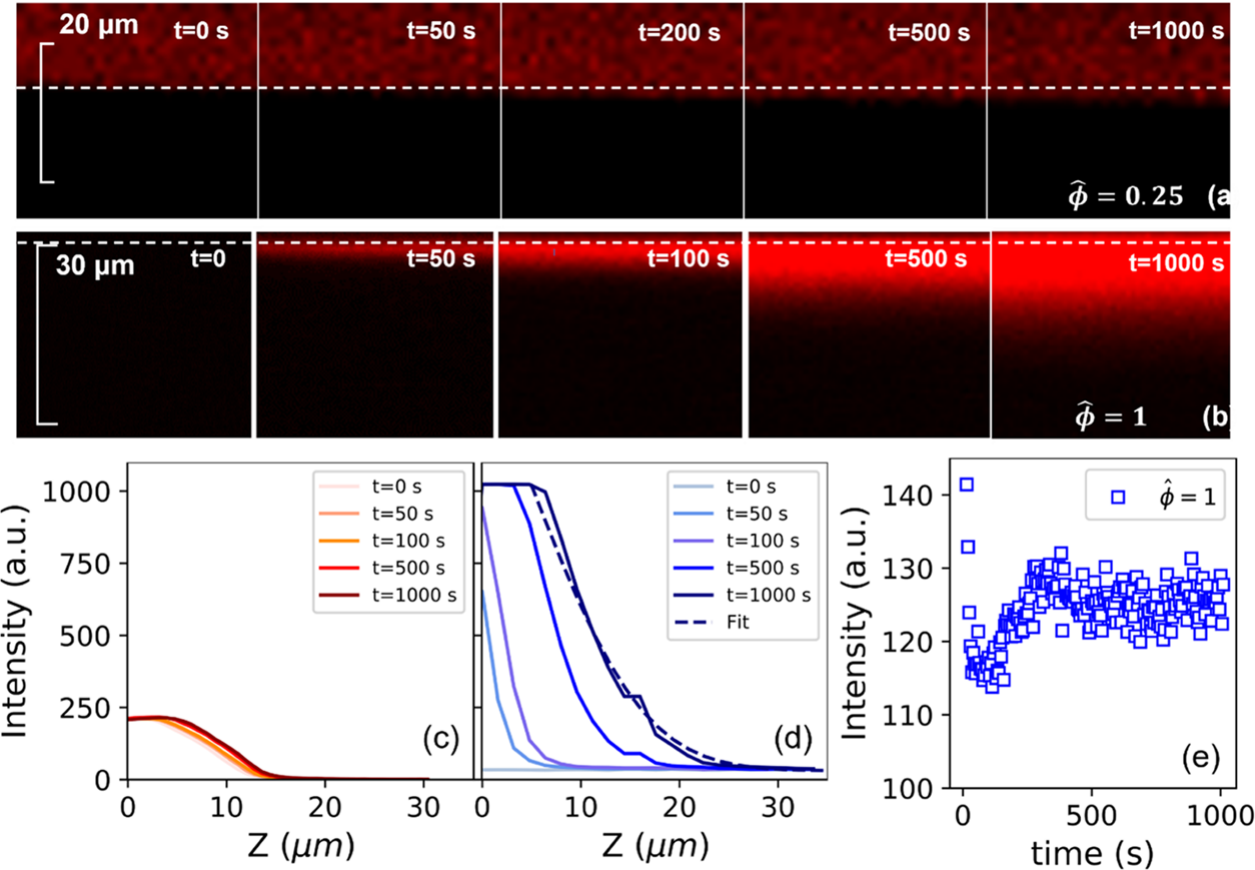
Diffusion of oil into the PSA over time. Evolution of the fluorescence signal with depth at different contact times for (a) ϕ̂ = 0.25 and (b) ϕ̂ = 1. The dotted line shows the approximate position of the interface between PDMS (above) and PDMS (below). The intensity of the fluorescence signal is plotted with depth from the interface at different contact times for (c) ϕ̂ = 0.25 and (d) ϕ̂ = 1. (e) The intensity of the fluorescence signal within fully swollen PDMS (ϕ̂ = 1) is plotted with contact time at depth *z* = −6.4 μm from the interface.

We use confocal microscopy to estimate the time scale for the diffusion of silicone oil from the PDMS into the PSA at two different oil fractions (Figure 6). As a control experiment, we looked at the intensity profile for fully swollen PDMS in contact with a flat glass surface and did not detect any oil film (Figure S2) but rather a sharp boundary between the PDMS and glass. We did not detect a fluid layer at the interface for ϕ̂ = 1 in contact with glass as well as ϕ̂ = 0.25, 1 in contact with PSA. For the trapped oil signal at the interface to go undetected, the thickness of the oil film must be smaller than the *z*-resolution of ∼0.8 μm of the laser scanner. This means that the fluid thickness at the interface must be below ∼0.8 μm, which agrees with the values reported by Lavielle et al. and Prieto-López et al.^9,10^ The optical properties of the PSA thin film allow us to visualize the diffusion of fluorescent dyed oil into the PSA. We assume that the lipophilic Nile red dye is well dissolved in silicone oil, and the intensity signal observed would also reflect oil transport across the interface into the PSA. For PDMS at ϕ̂ = 0.25 in contact with PSA, there is no indication of oil transport into the PSA from the interface since the intensity profile over time (Figure 6c) remains constant with time in both the PDMS and the PSA layer. In contrast, for adhesion to fully saturated PDMS (ϕ̂ = 1), we see a growing oil front that seeps into the PSA over time (Figure 6b). We observe a relatively low signal intensity within the PDMS in Figure 6e due to the scattering of light within the PDMS. We can also obtain an effective diffusivity of the oil into the PSA, assuming that the intensity profiles follow the concentration profile for diffusion into a semi-infinite medium (eq 2).^46^
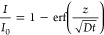
2

By fitting the intensity data to eq 2, we obtain the diffusivity *D* of fluorescently dyed silicone oil in PSA. *D* = 1.6 × 10^–13^ m^2^/s is 3 orders of magnitude smaller than the oil diffusivity in PDMS based on poroelastic relaxation data^15^ and swelling^11^ studies. Since the diffusivity is calculated based on dye fluorescence and transport, it is possible for oil diffusivity to be different from the value reported here. Based on the small size of the dye molecule (318 g/mol) compared to the oil (1240 g/mol), we can argue that the oil would diffuse more slowly than the dye. Even at full saturation, we see that the oil diffuses by only ∼2 μm by *t* = 100 s. Here, it is important to note that even though the diffusion length scale seems significant when compared to the fluid thickness at the interface, the effective volume fraction of oil in the PSA would be extremely low due to the poor miscibility of the acrylic PSA and PDMS.

### Contact Aging of Fluid-Infused Surfaces

**Figure 7:**
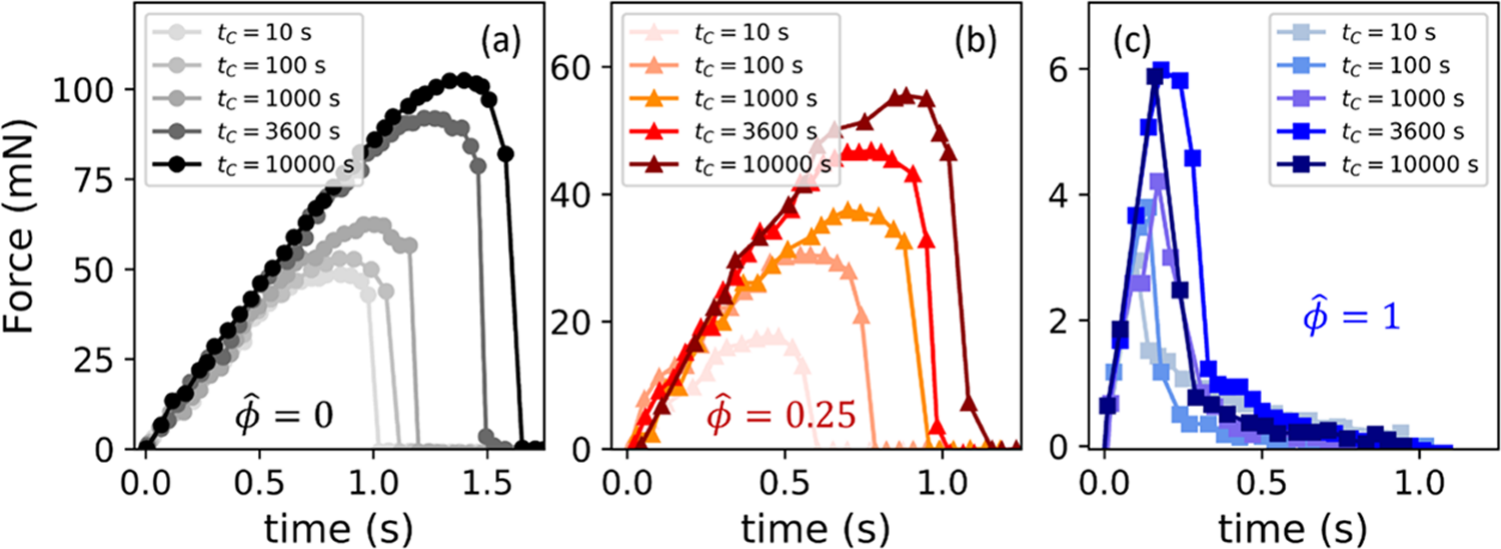
Maximum force during debonding increases with contact time at all oil fractions. Representative force vs time during detachment after a dwell at constant indentation for a contact time *t*_C_ are shown for (a) Dry PDMS (ϕ̂ = 0), (b) partially swollen PDMS (ϕ̂ = 0.25), and (c) fully saturated (ϕ̂ = 1). Force vs time curves at each contact time are representative curves selected from a set of 3 repeats (reported in Figure S3).

We investigate the effect of contact time and load relaxation on adhesion between the swollen PDMS and PSA. We measure adhesion during detachment of the PDMS probes from the PSA across a wide range of contact times, *t*_C_ (Figure 7). These curves are obtained after load relaxation at constant indentation δ ∼ 37 μm across the full range of oil volume fraction, as well as after a constant dwell force of *F* = 10 mN (Figures S7 and S8). The indentation depth is optimized to ensure that poroelastic and viscoelastic relaxation processes are significantly decoupled and can be observed within the time frame of a day.^15^ We see that the adhesive strength increases with contact time for dry, partially swollen, and fully swollen PDMS. Only for detachment with a fully swollen probe do we observe a capillary bridge. For adhesion with a dry probe, the adhesive strength increases by a factor of ∼2.5 for contact time between *t*_C_ = 10^2^ and 10^3^ s (Figure 7a), while it increases by a factor of ∼1.6 across the same contact time for partially swollen PDMS (Figure 7b). For the fully saturated PDMS, we observe a similar relative increase in adhesive strength, although the magnitude of the adhesive strength and its change with time is quite small (Δ*F*_max_ ∼ 3.5 mN) compared to the dry or partially swollen PDMS; therefore, any reported increase should be treated with caution.

**Figure 8:**
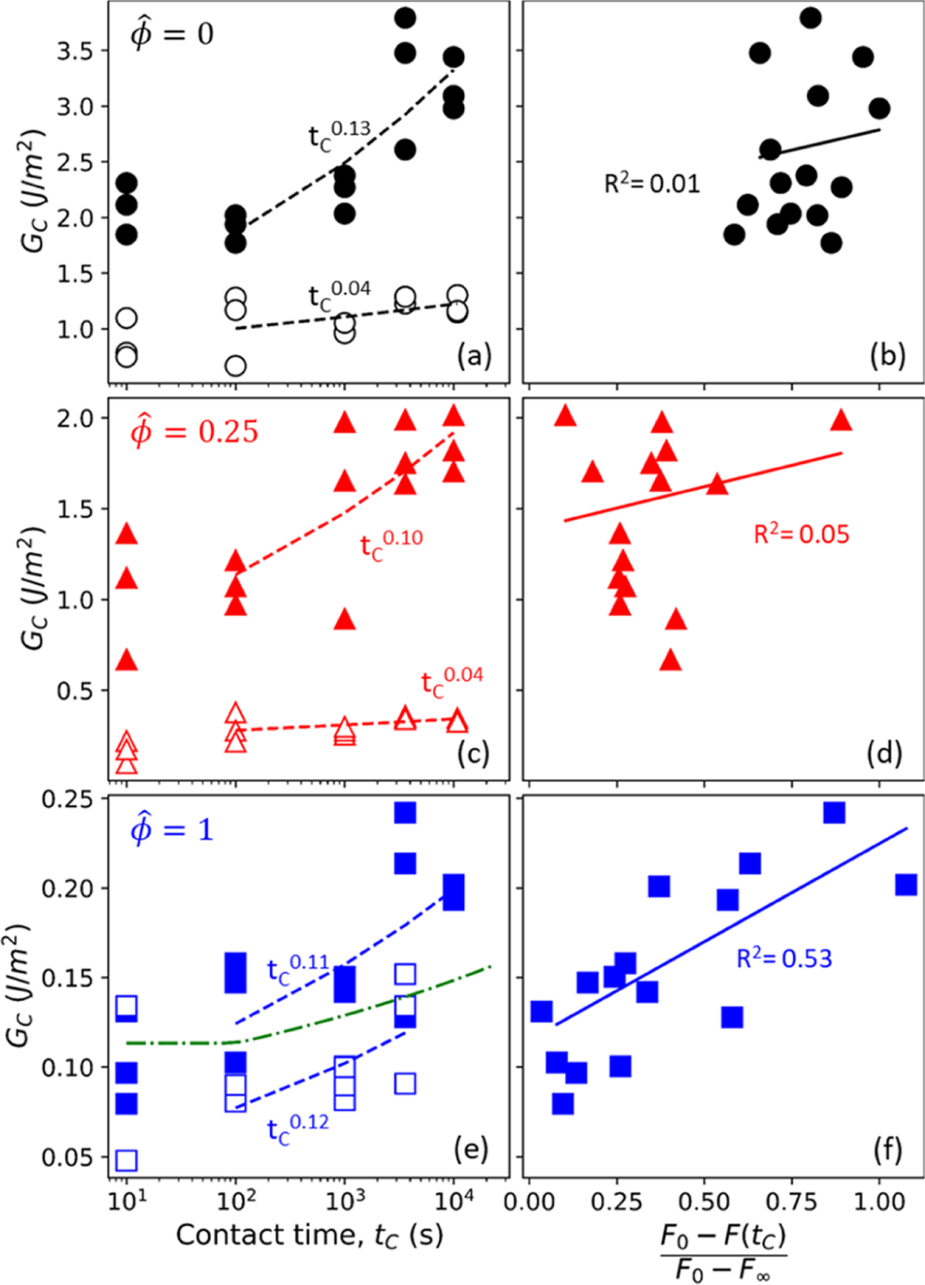
Comparison between contact aging and PDMS relaxation. The left column shows *G*_C_ as a function of contact time for contact between PDMS probes and either PSA or glass for (a) ϕ̂ = 0, (c) ϕ̂ = 0.25, and (e) ϕ̂ = 1. The right column shows *G*_C_ plotted as a function of the extent of relaxation (constant indentation δ = 37 μm) for (b) ϕ̂ = 0, (d) ϕ̂ = 0.25, and (f) ϕ̂ = 1. Filled symbols denote adhesion with PSA, and open symbols denote adhesion with glass. Dashed lines in (a), (c), and (e) represent power-law dependence. The dotted dashed green line in (e) is obtained from the capillary adhesion model (Supporting Information). Lines in parts b, d, and (f) are obtained using a linear fit between *G*_C_ and extent of relaxation (*F*_0_ – *F*(*t*_C_))/(*F*_0_ – *F*_∞_).

Contact aging occurs faster for the PDMS–PSA contact compared to the PDMS–glass contact (Figure 8). We obtain a critical strain energy release rate (*G*_C_) from JKR theory from the pull-off force at different contact times. We assume a power-law function, *G*_C_ ∝ *t*_C_^*n*^ for *t*_C_ > 100 s, to compare the adhesion of PDMS in contact with the PSA to the contact with the glass for the different swelling ratios.^15^ Power-law dependence has been frequently used in the literature to indicate an increase in adhesion over contact times spanning multiple decades.^20,47,48^ The power-law exponents given in Table 1 have been obtained using a nonlinear least-squares fit and can have significant variability as indicated by the standard error, especially for saturated PDMS contact with glass/PSA (Figure 8e). For the dry (ϕ̂ = 0) and partially swollen (ϕ̂ = 0.25) PDMS, adhesion with the PSA is not just higher in magnitude than adhesion to glass, but the contact also ages faster for the PDMS/PSA contact (adhesion builds up faster). From Table 1, *n* = 0.13 ± 0.03 for dry PDMS/PSA adhesion compared to *n* = 0.04 ± 0.03 for dry PDMS/glass adhesion (indicating negligible contact aging with glass) within reasonable limits of error. The more pronounced contact aging for the PDMS/PSA pair could be due to either (1) more pronounced stress relaxation^49−51^ or (2) chain interpenetration with the PDMS due to the contact between two polymeric interfaces.^23,24,52,53^

**Table 1:** Power-Law Exponent *n* for *G*_C_ vs Contact Time Data Shown in Figure 8

ϕ̂	*n*_PDMS/PSA_	*n*_PDMS/glass_
0	0.13 ± 0.03	0.04 ± 0.03
0.25	0.10 ± 0.04	0.04 ± 0.02
1	0.11 ± 0.03	0.12 ± 0.06

We compare the increase in adhesion with contact time to the extent of relaxation during dwell, defined as (*F*_0_ – *F*(*t*_C_))/(*F*_0_ – *F*_∞_) (Figure 8b,d,f). Here, the extent of relaxation is defined as the fraction of total relaxation at a given contact time *t*_C_, where *F*_0_ is the initial load at time *t* = 0, *F*(*t*_C_) is the load at contact time *t* = *t*_C_, and *F*_∞_ is the equilibrium load at time *t* → ∞ (see the Supporting Information for more details on how the extent of relaxation is obtained). Below, we discuss contact aging for the three different PDMS probes (dry, ϕ̂ = 0.25, and fully saturated).

For contact with the dry PDMS (ϕ̂ = 0), we see an increase in adhesion beyond *t*_C_ = 100 s, whereas the load relaxation reaches its steady state value around *t* ∼ 100 s. Therefore, an increase in adhesion beyond the viscoelastic relaxation time scale for the PDMS/PSA adhesion cannot be attributed to the viscoelastic load relaxation of the bulk materials. Instead, contact aging could be due to the strengthening of PDMS/PSA contact at the interface via chain interpenetration. The time scale over which we start to see an increase in adhesion, O(100 s), is also consistent with time scales reported in the literature for chain interpenetration.^52,54,55^ We also repeated the measurements, but this time, contact was maintained at a constant but lower load (Figures S9 and S10). Contact aging at constant load follows a similar trend for the PDMS/PSA pair (*n* = 0.09 ± 0.01, Table S3). However, for the glass/PDMS pair, contact aging seems to be faster for constant load (*n* = 0.09 ± 0.03) than for constant indentation measurements (0.04 ± 0.03). This could be due to small oscillations imposed by the control loop to maintain constant load, altering the contact formation with glass. The viscous nature of the soft PSA greatly dampens these oscillations, effectively minimizing the effect of the oscillations. A similar time dependence for contact aging between PDMS/PSA under constant load and constant indentation depth points to a similar mechanism, which is likely chain interpenetration.

Partially swollen (ϕ̂ = 0.25) PDMS/PSA adhesion follows a comparable contact aging to the one observed for the dry PDMS/PSA adhesion (*n* ≈ 0.1, Table 1). Due to the presence of oil in the network, both poroelastic and viscoelastic relaxation in the network contribute to overall load relaxation for contact with the partially swollen PDMS (see the Supporting Information). However, we do not observe any correlation between adhesion and relaxation for the partially swollen PDMS (Figure 8d), indicating that relaxation (including relaxation due to fluid transport within the network) does not contribute significantly to increasing the solid–solid contact. The similar aging exponent also hints toward a similar mechanism of chain interdiffusion for an increase in adhesion for dry and partially swollen PDMS (Figure 8a,c).

Contact aging with PDMS fully saturated with oil (ϕ̂ = 1) is much less significant (or measurable), especially as adhesion is much lower. At short contact times (*t*_C_ ≤ 100 s), adhesion to either PSA or glass remains within the limits of capillary adhesion. For longer contact times, PDMS/PSA adhesion seems to increase beyond the estimate from the capillary adhesion model (Figure 8e) and increases from its initial value of *G*_C_ = 0.14 ± 0.02 J/m^2^ at *t*_C_ = 100 s to *G*_C_ = 0.199 ± 0.003 J/m^2^ at *t*_C_ = 3600 s. While the capillary adhesion model overestimates the adhesion with glass, the prediction for PSA–PDMS adhesion is underestimated if they are only attributed to capillarity. Based on contact angle measurements (Figure 5), increasing the oil fraction in the PDMS limits initial solid–solid contact (the PDMS is almost fully covered with oil, ϕ_S_ ∼ 0.82 ± 0.17, at ϕ̂ = 0.55). The capillary adhesion model for swollen PDMS–PSA adhesion would only account for the aging of existing solid–solid contact. Here, it underestimates adhesion at higher contact times and is not sufficient to capture the increased adhesion. In addition, the increase in adhesion with contact time (Figure 8e) has a weak correlation with the extent of relaxation (Figure 8f). Load relaxation for the fully swollen PDMS happens over a time scale consistent with poroelastic relaxation caused by fluid transport within the PDMS (Figure S5). We also see an increase in adhesion over the poroelastic time scale τ_P_ = 965 ± 271 s, which could indicate that fluid transport within the PDMS may contribute to better solid–solid contact for a saturated PDMS. In contrast, we see a negligible increase in adhesion between PDMS and PSA at ϕ̂ = 0.6 and ϕ̂ = 1 at constant load over a time scale of *t*_C_ ∼ 10^4^ s (Figure S9c,d). Since swollen PDMS at constant load is not allowed to relax, it stands to reason that poroelastic relaxation and subsequent fluid transport would not contribute to the increase in adhesion at constant load. However, due to very low adhesion with saturated PDMS, we cannot draw any substantial conclusions regarding the precise role of poroelasticity-driven fluid transport in affecting adhesion.

**Figure 9:**
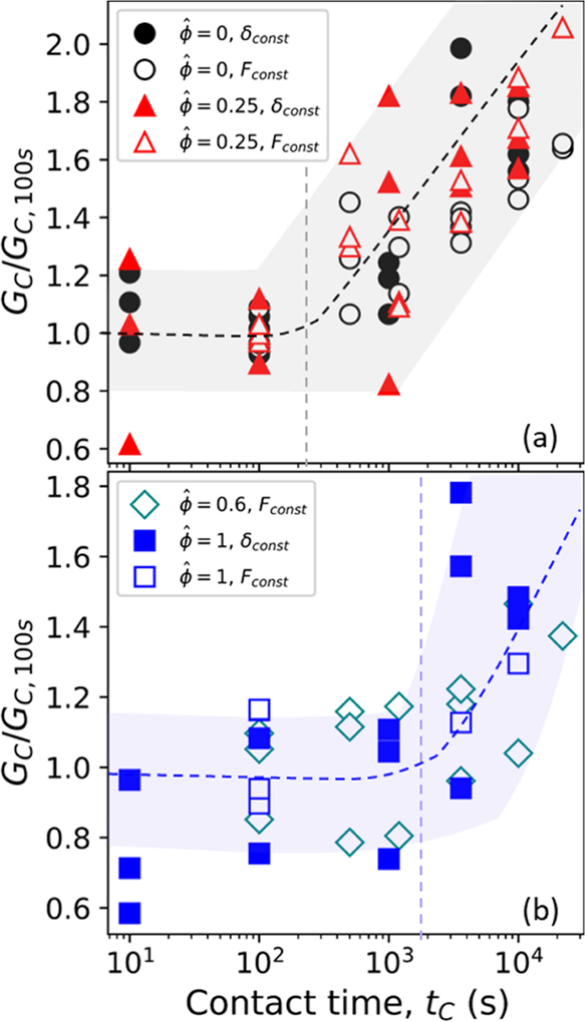
Delay in the onset of contact aging. The critical strain energy release rate normalized with its value at *t*_C_ = 100 s as a function of contact time for (a) ϕ̂ = 0 and ϕ̂ = 0.25 and (b) ϕ̂ = 0.6 and ϕ̂ = 1. The shaded region with the dashed line is drawn to guide the eye.

We see a shift in the onset of contact aging for PDMS/PSA contact when the oil fraction in PDMS is high. We define the onset of contact aging as the contact time beyond which adhesion starts increasing beyond 25% of its initial value at *t*_C_ = 100 s. In Figure 8a,c,e, we observe that the time of onset at ϕ̂ = 0 and ϕ̂ = 0.25 is O(100 s), whereas the time of onset at ϕ̂ = 1 is O(1000 s). This shift in the onset of contact aging can be visualized if we plot *G*_C_ normalized with *G*_C,100 s_ as a function of contact time (Figure 9). The time of onset is nearly independent, O(100 s), of the load, as it is similar across constant indentation and constant load experiments for dry and partially swollen PDMS. Adhesion to a swollen PDMS probe can increase over time through the contact aging process of chain interdiffusion and the formation of new solid–solid contact. We established previously that chain interpenetration is the likely mechanism for increasing adhesion for dry and partially swollen PDMS with PSA. For the fully swollen PDMS/PSA system undergoing relaxation at constant indentation prior to detachment, adhesion increases around the poroelastic relaxation time scale-hinting at the possibility that fluid removal from the interface could play a role in delaying solid–solid contact formation and increase in adhesion.

The fluid near the interface can be removed via three different processes: (1) poroelasticity-driven fluid transport away from the contact region, (2) hydrodynamic drainage away from the interface, and (3) diffusion of fluid into the PSA. We previously established that poroelastic relaxation may play a role in increasing adhesion for fully saturated PDMS in contact with PSA at constant indentation depth. In addition, drainage of fluid trapped within the contact region or diffusion of oil into PSA could both remove oil from the contact region, leading to an increase in adhesion. We can obtain an estimate for the drainage time necessary to reach a given fluid film thickness using the Stefan–Reynolds^42^ model (see the Supporting Information, Figure S11). We find that given an onset for contact aging of *t*_C_ ∼ 1000 s, the fluid gap at the interface would reduce from its initial value around O(100) to O(10) nm over that time period. In contrast, fluid would diffuse into the PSA by , which is around half the thickness of the PSA layer. Fluid diffusion into the PSA is a much slower process and would contribute to fluid removal over much longer time scales. Fluid drainage is also quite slow and would necessitate a fluid film that is thinner than the polymer chains to facilitate bridging. For adhesion with swollen PDMS near saturation at constant load, diffusion into PSA and hydrodynamic drainage could contribute to fluid removal and a subsequent increase in adhesion. It is important to note here that our arguments for the underlying mechanism for fluid removal are based on simple approximations. While the effect of the presence of oil at the interface in delaying adhesion is relatively clear from the data, additional studies will be necessary to determine the different mechanisms.

## Conclusions

We aimed to investigate the effect of oil in swollen elastomers on adhesion with a soft adhesive surface. We looked at the adhesion of swollen PDMS with an acrylic PSA at different contact times for a range of oil content. We found that solid–solid contact formation is better between PDMS and PSA as compared to that between PDMS and glass. A comparison of the effective surface fractions for the PDMS surface and PDMS/PSA contact suggests that PSA can maximize contact with the existing solid PDMS surface. Mechanistically, better contact and stronger adhesion at short times are consistent with the compliant behavior of the PSA and its ability to form good conformal contact with rough surfaces (or here with surfaces partially covered with oil). This ability to be able to seek out solid–solid contact is analogous to the ability of mussel feet to find a solid area on the swollen elastomer. However, a key difference is the absence of any active sensors on the surface of the PSA.

We also looked at the effect of the oil fraction over long contact times on adhesion with swollen elastomers. We found that while PDMS was able to maintain its antiadhesive properties with glass over long contact times, this ability was diminished in the presence of a PSA. The time scale for contact aging was consistent with the time scale associated with polymer interdiffusion at the interface. As PDMS becomes saturated with oil, the onset of contact aging is delayed and weakly correlates with polymer relaxation. We also found that the amount of oil in the bulk of the PDMS plays a key role in maintaining a low adhesion for longer periods of time, even on the PSA surface. We demonstrated that the amount of fluid in the elastomer affects the time-dependent increase in adhesion or contact aging. Excess fluid at the interface is necessary but not sufficient to reduce the adhesion with highly adhesive surfaces. Strategies to maintain low adhesion over time on sticky surfaces could be the subject of future investigation.

Combined, our results show that soft adhesive surfaces behave differently when in contact with swollen elastomers. Fluid content within the elastomer and contact time both play important roles in setting the adhesion between the two surfaces. While the presence of fluid hinders the adhesion of the soft adhesive with the elastomer [as is the case with slippery liquid-infused porous surfaces (SLIPS)], the adhesion can be recovered with time. The time effect of adhesion is also coupled with the effect of oil content in which excess fluid at the interface tends to win in the competition to affect adhesion.

## Supporting Information Available

(1) confocal imaging of the contact region; (2) poroelastic relaxation, (3) capillary adhesion model, (4) detachment at constant load, and (5) Stefan–Reynolds model (PDF)
