# Supplementary material for: Adhesion and Contact Aging of Acrylic Pressure-Sensitive Adhesives to Swollen Elastomers
Source: Langmuir. 2024 Feb 15;40(8):4267–76. doi: 10.1021/acs.langmuir.3c03413 (PMC10906000; doi:10.1021/acs.langmuir.3c03413)
Supplement: Supplementary file 1 — la3c03413_si_001.pdf [file la3c03413_si_001.pdf]

<u>**SUPPORTING INFORMATION**</u>

Adhesion and contact aging of acrylic pressure sensitive adhesives to swollen elastomers

Anushka Jha[1], Stefan Gryska[2], Carlos Barrios[3], and Joelle Frechette[4,5,*]

[1]Chemical and Biomolecular Engineering, Johns Hopkins University, Baltimore, Maryland 21218, United States
[2]3M Company, 3M Center, Building 201-4N-01, St. Paul, Minnesota 55144-1000, United States
[3] Carlos Barrios Consulting LLC, Frisco, Texas 75034, United States
[4]Chemical and Biomolecular Engineering, University of California, Berkeley, California 94720, United States
[5]Lawrence Berkeley National Laboratory, Energy Technology Area, Berkeley, California 94720, United States

*Corresponding author: jfrechette@berkeley.edu

Number of pages 10
Number of figures: 11
Number of tables: 4

1. Confocal Microscopy.

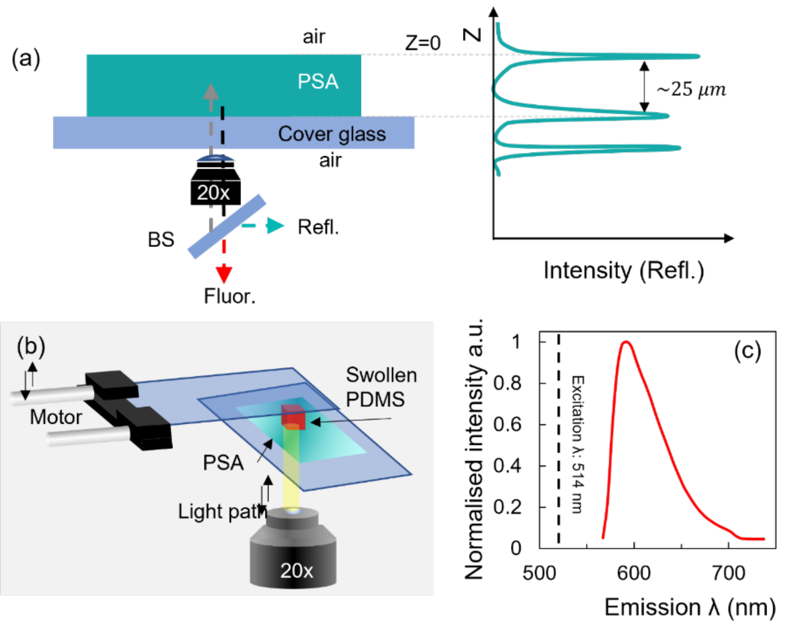

Fig. S1. Confocal microscopy set-up. (a) Locating the top surface of the PSA using reflected light. The beam splitter (BS) splits the output signal into fluorescence(fluor.) and reflectance(refl.). Intensity of reflected light is measured as a function of depth in z-direction. The first two intensity peaks from the top are ~25 μm apart, which is around the thickness of the PSA. (b) Schematic of swollen PDMS in contact with PSA. (c) Normalized intensity vs. emission wavelength of swollen

PSA infused with fluorescent silicone oil. The maximum in the intensity curve occurs at $\lambda = 561\ nm$.

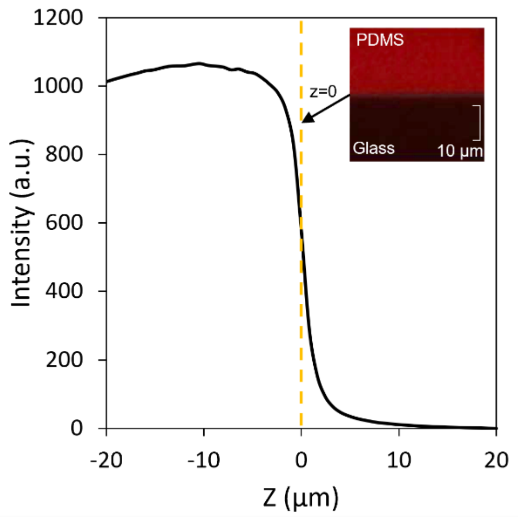

Fig. S2. Fluorescence intensity as a function of depth for a swollen PDMS ($\hat{\phi} = 1$) in contact with glass.

2. Repeat of adhesion measurements

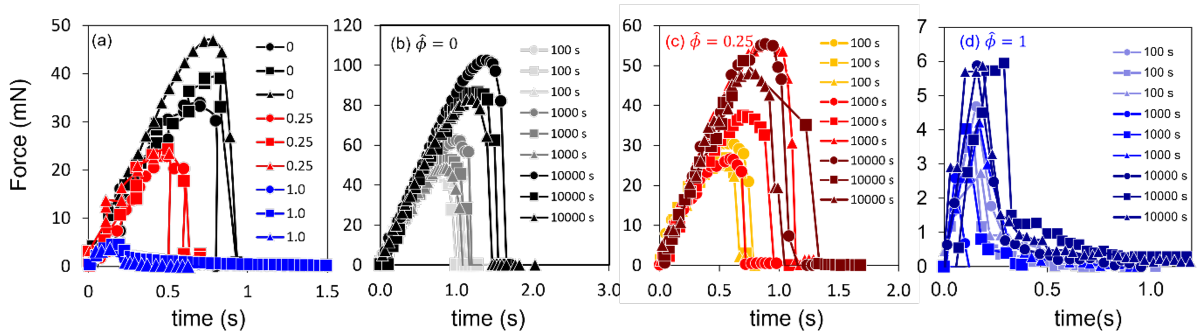

Fig. S3. Debonding curves for PDMS-PSA adhesion after contact at (a) constant load F = 10 mN for 100 s, (b-d) at constant indentation depth $\delta = 37\mu m$ for three different contact times contact time t_c=100 s, 1000 s and 10000 s.

3. Relaxation during constant indentation (effect of viscoelasticity)

During constant indentation, the load F in the PSA – PDMS system relaxes over time for all $\hat{\phi}$. The relaxation curves can be fit to a poro-viscoelastic model to obtain the overall viscoelastic and poroelastic timescales in the system.

$$F(t) = (F_0 - F_V)\,exp\left(-\frac{t}{\tau_V}\right)^{\beta} + (F_V - F_P)exp(-t/\tau_P) + F_P. \quad \text{(S1)}$$

Where F_0 is maximum compressive load on the system that corresponds to $t = 0$. F_V and F_P are the loads after viscoelastic and poroelastic relaxation. **Eq. (S1)** is valid for a system where the poroelastic and viscoelastic timescales are sufficiently different i.e., $\tau_V \ll \tau_P$ which is indeed the case for our constant indentation experiments. (**See Table S1**). For dry PDMS **Eq. (S1)** reduces to a stretched exponential relaxation

$$F(t) = (F_0 - F_V)\, exp(-t/\tau_V)^{\beta} + F_V \tag{S2}$$

Eq. (S2) is an alternate mathematical expression for the Prony series.[1] A dispersion factor $\beta <$ 1 indicates that the system cannot be described using a single exponential relaxation time, but rather a distribution of timescales $0 < \tau_V < \infty$. The contribution of each timescale in the distribution depends on β. The mean characteristic time for viscoelasticity $\langle \tau_V \rangle$ can be calculated for the stretched exponential distribution using **Eq. (S3)**.

$$\langle \tau_V \rangle = \int_0^{\infty} dt\, e^{-\left(\frac{t}{\tau_V}\right)^{\beta}} = \frac{\tau_V}{\beta} \Gamma\left(\frac{1}{\beta}\right) \tag{S3}$$

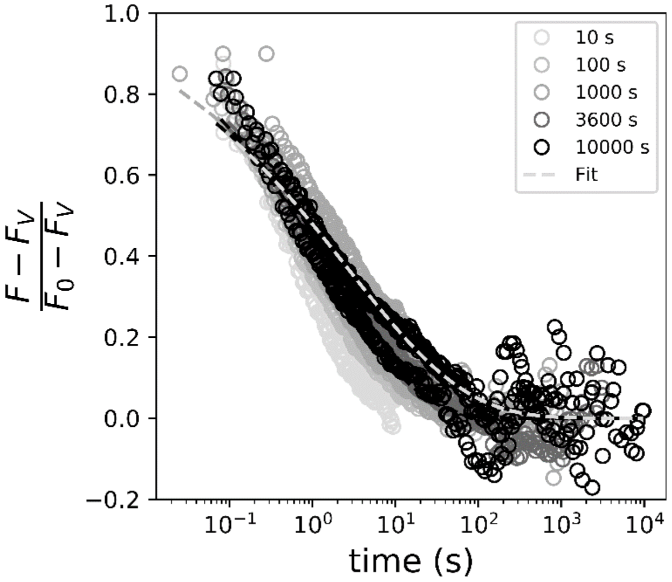

Fig. S4. Normalized load $(F - F_V)/(F_0 - F_V)$ vs. contact time during load relaxation of dry PDMS in contact with PSA at constant indentation $\delta \sim 37\ \mu m$.

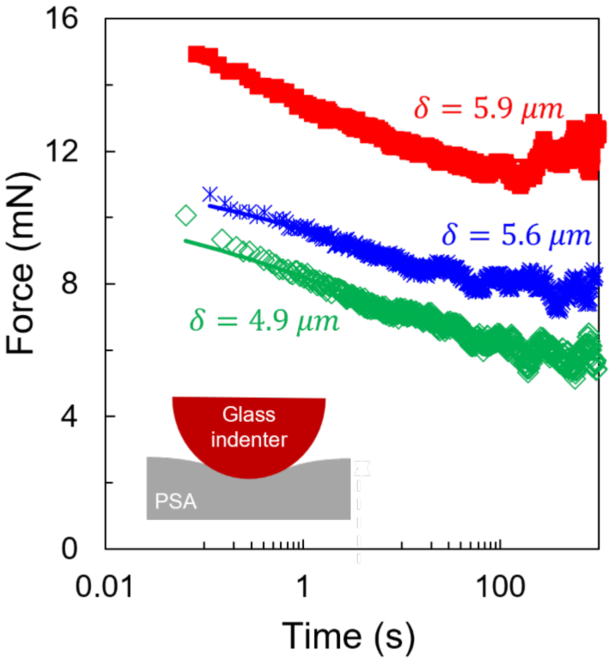

Fig. S5. Load relaxation during indentation of PSA in contact with a glass indenter. The nominal stress on the material $\sigma = F/A$ is maintained at 91 kPa.

In **Fig. S3** we see that most of the load on the dry PDMS has relaxed after 100 s, which is similar to the relaxation of PDMS on glass.[2] Therefore, the bulk viscoelastic relaxation in the PDMS-PSA system is governed by the relaxation of the PDMS. It can be seen in **Fig. S4** that for the same nominal stress, the PSA undergoes an indentation of $\sim 6\ \mu m$ with a glass probe. The presence of PDMS in the system would further suppress bulk deformation in the PSA as the load will be distributed between the PSA and PDMS.[3]

4. Capillary adhesion model

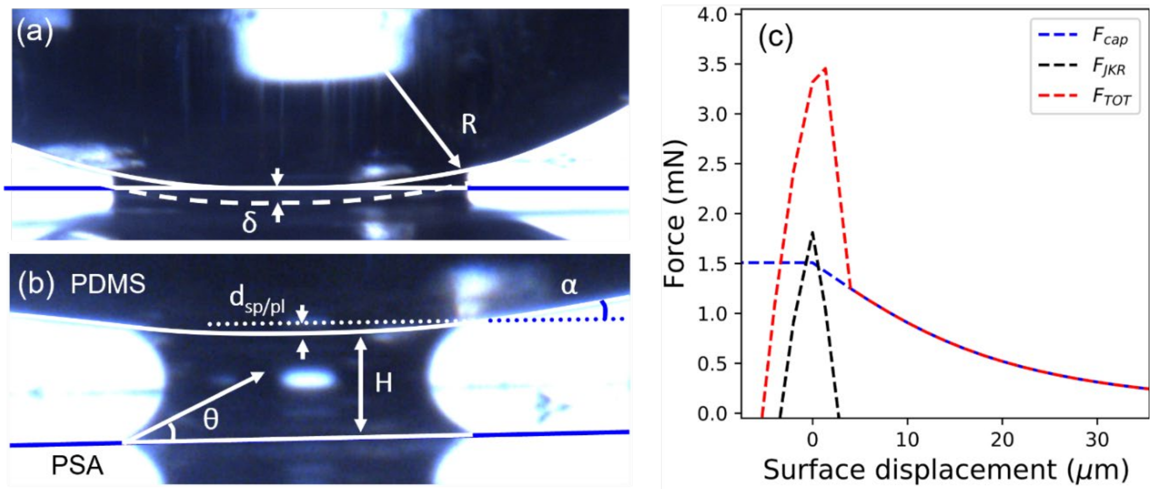

Fig. S6. Capillary adhesion model for swollen PDMS adhesion with PSA. Side view image of fully saturated PDMS in contact with PSA (a) before jump-off – both JKR and capillary forces act on the PSA-PDMS system. (b) After jump-off – liquid capillary bridge formed at the interface contributes to total attractive force. (c) Individual contributions of JKR adhesive force F_{JKR} and capillary force F_{cap} and total contribution F_{TOT} calculated using Eq. S4-S6.

$$F_{JKR} = -\frac{4}{3}E^*a^3 - 2\sqrt{2\pi w E^* a^3}; \delta = \frac{a^2}{R} - \sqrt{\frac{2\pi w a}{E^*}} \quad \text{(S4a)}$$

$$F_{cap} = -4\pi\gamma_{oil}R \quad \text{(S4b)}$$

$$F_{cap} = -\frac{4\pi\gamma_{oil}R\cos\theta}{1 + \frac{H}{d\frac{sp}{pl}}} - 2\pi\gamma_{oil}R\sin(\theta + \alpha) \quad \text{(S5)}$$

$$F_{TOT} = F_{cap} + F_{JKR} \quad \text{(S6)}$$

In Eq. (S4a), $w = w_0\left(\frac{t_C}{t_{ref}}\right)^n$ where $n = 0.13$ is the contact aging exponent for dry contact, $t_{ref} = 100\ s$, and $w_0 \sim 60$ mJ/m^2 is the thermodynamic work of adhesion for dry PDMS contact at $t_C = t_{ref}$.

5. Relaxation during constant indentation (effect of oil transport)

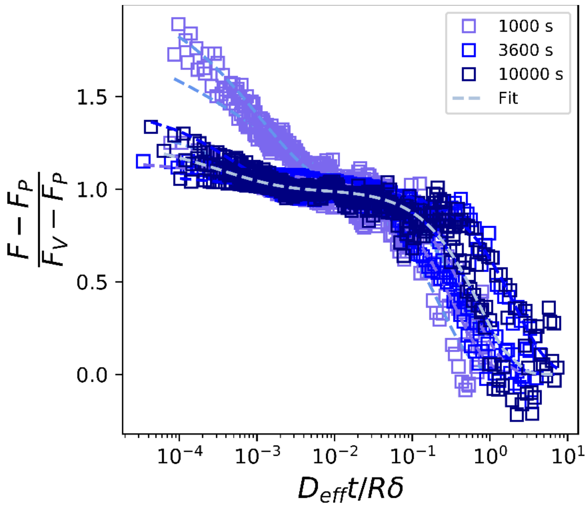

Fig. S7. Normalized load $(F - F_P)/(F_V - F_P)$vs. contact time during load relaxation of fully swollen PDMS ($\hat{\phi} = 1$) in contact with PSA at constant indentation $\delta \sim 37\ \mu m$.

For swollen PDMS in contact with PSA (**Fig. S5**), we find that the effective diffusivity obtained (see **Table S1**) is very similar to that for glass-PDMS[2] indicating that oil diffusion into the PSA is has either negligible contribution to relaxation and/or happens over a much different timescale.

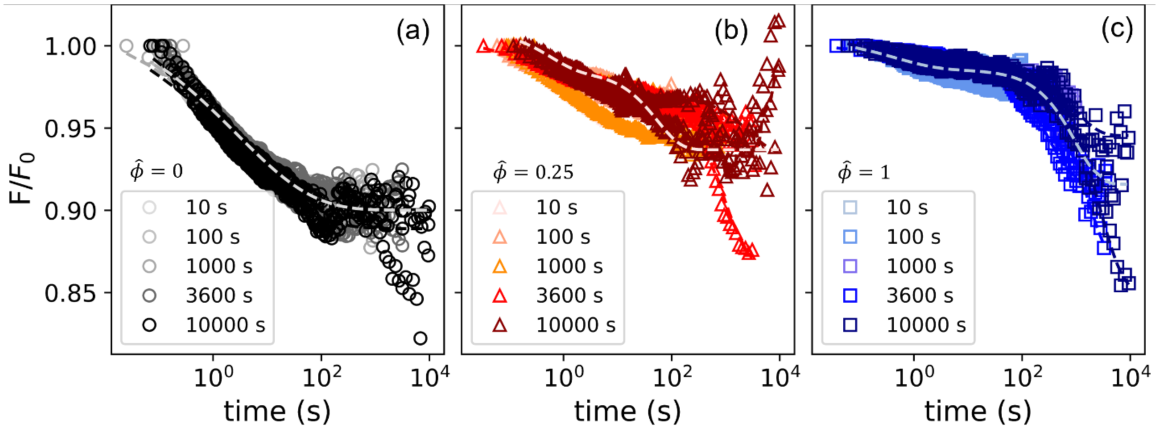

Fig. S8. Normalized load (F/F_0) vs. contact time during load relaxation at constant indentation $\delta \sim 37\ \mu m$. (a) $\hat{\phi} = 0$, (b) $\hat{\phi} = 0.25$, (c) $\hat{\phi} = 1$.

Table S1. Poro-viscoelastic relaxation parameters obtained by fitting Eq. S4 and Eq. S5 to load relaxation data.

$\hat{\phi}$	$\tau_V\ (s)$	β	$\delta\ (\mu m)$	$D_{eff}\ (\times 10^{10}\ m^2/s)$	$\tau_P\ (s)$
**0**	1.9 ± 0.9	0.4 ± 0.1	34 ± 3	$--$	$--$
**0.25**	0.9 ± 0.2	0.47 ± 0.03	36 ± 4	15.4 ± 6.6	903 ± 423
**1**	1.0 ± 1.1	0.7 ± 0.1	38 ± 2	3.7 ± 0.9	956 ± 271

Estimating extent of relaxation:

The extent of relaxation $\frac{F_0 - F(t_C)}{F_0 - F_\infty}$ is defined as the fraction of total relaxation at a given contact time t_C, where F_0 is the initial load at time $t = 0$. $F(t_C)$ is the load at contact time $t = t_C$, and F_∞ is the equilibrium load at time $t \rightarrow \infty$.

We obtain $F(t_C)$ as the average of the load for the last n seconds where the slope of the force time curve becomes constant (within 1%). Typically, n~ 20% of the total contact time.

Since we do not know F_∞ for an indented sample a priori, we assume that all samples at the same oil content will reach the same state of equilibrium and estimate F_∞ to be the minimum obtained value of $F(t_C)$. To correct for variations across experiments, we rescale with the initial load to obtain F_∞/F_0. (Starred value in **Table S2** below). The extent of relaxation can therefore be rewritten as $\frac{1 - F(t_C)/F_0}{1 - F_\infty/F_0}$.

Table S2. Experimental data used to obtain extent of relaxation in **Fig. 8 (b, d, f)**.

$\hat{\phi}$	Contact time, t_c (s)	F_0 (mN)	$F(t_C)$ (mN)	$F(t_C)/F_0$	$\frac{F_0-F(t_C)}{F_0-F_\infty}$ or $\frac{1-F(t_C)/F_0}{1-F_\infty/F_0}$
0	10	68.8	63.1	0.92	0.59
	10	73.7	66.2	0.90	0.72
	10	77.8	70.9	0.91	0.62
	100	65.9	59.3	0.90	0.71
	100	61.4	54.3	0.88	0.82
	100	63.8	56.1	0.88	0.86
	1000	64.4	57.2	0.89	0.79
	1000	59.9	52.3	0.87	0.89
	1000	61.9	55.3	0.89	0.75
	3600	73.4	65.1	0.89	0.80
	3600	67.6	61.3	0.91	0.66
	3600	71.7	64.8	0.90	0.69
	10000	70.3	60.9	0.87	0.95
	10000	71.6	61.5	**0.86****	1.00
	10000	72.2	63.8	0.88	0.82
0.23	10	56.7	53.5	0.94	0.45
	10	60.7	58.5	0.96	0.29
	10	56.9	54.9	0.96	0.29
	100	46.1	44.4	0.96	0.30
	100	56.1	53.9	0.96	0.31
	100	55.0	53.0	0.96	0.29
	1000	61.8	58.2	0.94	0.47
	1000	59.4	56.2	0.95	0.42
	1000	84.7	80.2	0.95	0.43
	3600	60.5	56.0	**0.92****	0.60
	3600	63.9	55.9	0.87	1.00
	3600	67.7	64.4	0.95	0.39
	10000	60.4	59.5	0.99	0.12
	10000	57.3	54.2	0.94	0.44
	10000	68.8	67.0	0.97	0.20
1	10	60.9	60.1	0.99	0.09
	10	76.7	76.3	1.00	0.03
	10	71.4	70.0	0.98	0.12
	100	78.1	75.1	0.96	0.25
	100	78.3	76.5	0.98	0.15
	100	61.7	61.0	0.99	0.07
	1000	64.8	62.4	0.96	0.24
	1000	69.8	67.4	0.97	0.23
	1000	65.5	62.4	0.95	0.31
	3600	46.3	42.1	0.91	0.59
	3600	63.6	58.4	0.92	0.54

	3600	81.2	71.2	**0.88****	0.81
	10000	61.7	56.8	0.92	0.53
	10000	71.7	67.9	0.95	0.34
	10000	67.6	57.3	0.85	1.00

**indicates value used for F_∞/F_0

6. Detachment after constant load F= 10mN

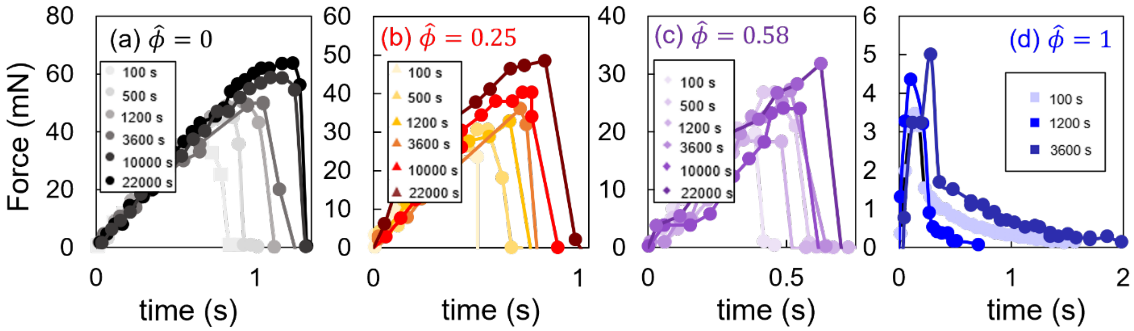

Fig. S9. force during debonding increases with time at all oil fractions. Force vs. time during detachment after constant dwell are shown for (a) Dry PDMS ($\hat{\phi} = 0$), (b) Partially swollen PDMS ($\hat{\phi} = 0.25$), (c) Partially swollen PDMS ($\hat{\phi} = 0.6$) and (d) Fully saturated ($\hat{\phi} = 1$). Liquid capillary bridge is present during detachment at all contact times for fully saturated PDMS.

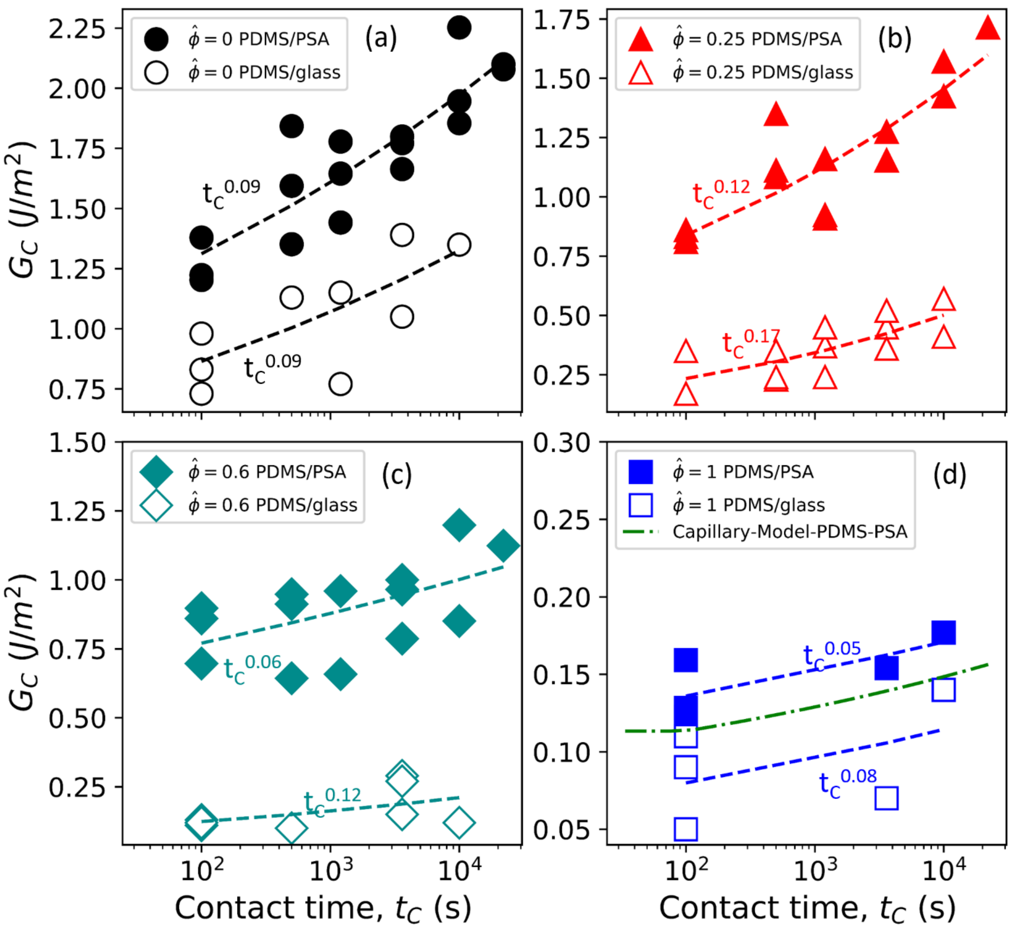

Fig. S10. Strain energy release rate as a function of time with PSA and glass for constant load F=10 mN experiments. (a) $\hat{\phi} = 0$, (b) $\hat{\phi} = 0.25$, (c) $\hat{\phi} = 0.6$, and (d) $\hat{\phi} = 1$. Filled symbols denote adhesion with PSA and open symbols denote adhesion with glass. Dashed lines represent power-law fit with contact time. Dotted dashed green line in (d) represents capillary adhesion model with contact aging.

Table S3. Power law exponents for strain energy release rate G_C vs contact time data shown in **Fig. S9.**

$\hat{\phi}$	PSA	Glass
0	0.09+/-0.01	0.09+/-0.03
0.25	0.12+/-0.02	0.17+/-0.05
0.58	0.06+/-0.02	0.12+/-0.09
1	0.05+/-0.02	0.08+/-0.08

7. Stefan-Reynolds equation for hydrodynamic drainage

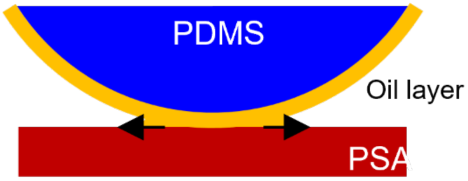

Fig. S11 Schematic describing hydrodynamic drainage between PSA and PDMS

Assuming drainage of fluid layer of thickness h follows Stefan Reynolds model[4] for dynamics of a thin fluid film draining under driving force F between two parallel surfaces, the rate of thinning of fluid layer can be expressed as

$$\frac{dh}{dt} = -\frac{2h^3}{3\pi\mu a^2} \cdot F \qquad \text{(S7)}$$

Where $\mu = 1.07\ Pa \cdot s$ is the fluid viscosity, a is radius of the confined region.

Integrating Eq. (S7), we get,

$$t = \frac{\tau_H h_0^2}{2}\left(\frac{1}{h^2} - \frac{1}{h_0^2}\right) \qquad \text{(S8)}$$

Where $\tau_H = \frac{3\pi\mu a^4}{2h_0^2 F}$ is the characteristic drainage timescale.

Table S4 Parameters for hydrodynamic drainage calculation (h_0~$0.4\ \mu m$)

	Constant indentation	Constant load
$F\ (mN)$	~70	10
$a\ (\mu m)$	~ 600	~300
$\tau_H(s)$	58	25

References

(1) Johnston, D. C., Stretched exponential relaxation arising from a continuous sum of exponential decays. *Phys Rev B* **2006,** *74*.

(2) Jha, A.; Karnal, P.; Frechette, J., Adhesion of fluid infused silicone elastomer to glass. *Soft Matter* **2022,** *18* 7579-7592.

(3) Sridhar, I.; Zheng, Z. W.; Johnson, K. L., A detailed analysis of adhesion mechanics between a compliant elastic coating and a spherical probe. *J Phys D Appl Phys* **2004,** *37* 2886-2895.

(4) Reynolds, O., IV. On the theory of lubrication and its application to Mr. Beauchamp tower's experiments, including an experimental determination of the viscosity of olive oil. *Philos. Trans. R. Soc. London* **1997,** *177* 157-234.
